# Tratamento endovascular do aneurisma aortoilíaco: relato do primeiro caso utilizando endoprótese brasileira com ramo ilíaco

**DOI:** 10.1590/1677-5449.011116

**Published:** 2017

**Authors:** Fábio Augusto Cypreste Oliveira, Carlos Eduardo de Sousa Amorelli, Fábio Lemos Campedelli, Davi Heckmann, Juliana Caetano Barreto, Maria Cunha Ribeiro Amorelli, Ana Flávia Guerra Campedelli, Philippe Moreira da Silva

**Affiliations:** 1 Hospital São Francisco de Assis, Serviço de Angiologia, Cirurgia Vascular, Endovascular e Laserterapia – AngioGyn, Goiânia, GO, Brasil.; 2 CENTERVASC-Rio, Cirurgia Vascular, Rio de Janeiro, RJ, Brasil.; 3 Universidade Federal de Goiás – UFG, Hospital das Clínicas, Infectologia, Goiânia, GO, Brasil.; 4 Instituto Nacional de Câncer – INCA, Hematologia e Hemoterapia, Goiânia, GO, Brasil.; 5 Hospital Araújo Jorge, Radioterapia, Goiânia, GO, Brasil.

**Keywords:** aneurisma da aorta abdominal, procedimentos endovasculares, artéria ilíaca

## Abstract

O aneurisma aortoilíaco tem representado desafio terapêutico principalmente em relação ao tratamento endovascular, visto que a embolização das artérias ilíacas internas pode levar a graves complicações. Inúmeras técnicas cirúrgicas convencionais e endovasculares têm sido descritas para a preservação de ao menos um ramo ilíaco interno. Dentre as opções de tratamento totalmente endovascular, podemos citar as endopróteses ramificadas e a técnica de próteses paralelas. Os autores relatam o primeiro caso de tratamento endovascular com preservação de ramo ilíaco interno utilizando endoprótese nacional ramificada.

## INTRODUÇÃO

A correção endovascular do aneurisma de aorta abdominal vem sendo aplicada em todo mundo, e a cada ano vem sendo observado um crescimento no número de casos^1^. O tratamento endovascular do aneurisma infrarrenal tem mostrado bons resultados em relação à exclusão aneurismática e à redução da morbimortalidade cirúrgica^2^. Porém, quando o aneurisma de aorta abdominal está associado ao aneurisma das artérias ilíacas, a dificuldade terapêutica se eleva, e o objetivo de excluir os aneurismas mantendo a circulação pélvica torna-se um desafio na tentativa de evitar a embolização bilateral da artéria ilíaca interna e, consequentemente, as complicações secundárias a essa oclusão^3^. De acordo com a literatura, os casos que necessitam de embolização uni ou bilateral das artérias ilíaca internas podem evoluir para claudicação glútea em 16-55% dos casos, seguida de impotência em 10-17% dos casos^4^^,^ 5. Complicações mais graves como isquemia medular/mesentérica ou necrose glútea são raras, mas podem acontecer em 1-3% dos casos de embolização bilateral^6^^,^ 7. Dessa forma, a tentativa de preservação da circulação pélvica através da manutenção do fluxo em pelo menos uma das artérias ilíacas internas vem sendo o objetivo de diversas técnicas de revascularização, tais como: revascularização por ponte/bypass^8^^,^ 9, utilização de endopróteses com ramos ilíacos^10^^,^ 11, e utilização de técnica de endopróteses em paralelo, como a técnica sanduíche descrita por Lobato^12^.

As endopróteses com ramo ilíaco (IBD, do inglês *iliac branch device*) foram desenvolvidas com o objetivo de promover tratamento totalmente endovascular dos aneurismas aortoilíacos, com exclusão aneurismática e manutenção do fluxo anterógrado das artérias ilíaca internas. Esses novos dispositivos vêm mostrando resultados iniciais favoráveis e promissores^11^^,^ 13 ^,^ 14.

O objetivo deste artigo foi descrever o primeiro caso de tratamento endovascular de aneurisma aortoilíaco utilizando endoprótese brasileira com ramo ilíaco.

## DESCRIÇÃO DO CASO

**Figura 1:**
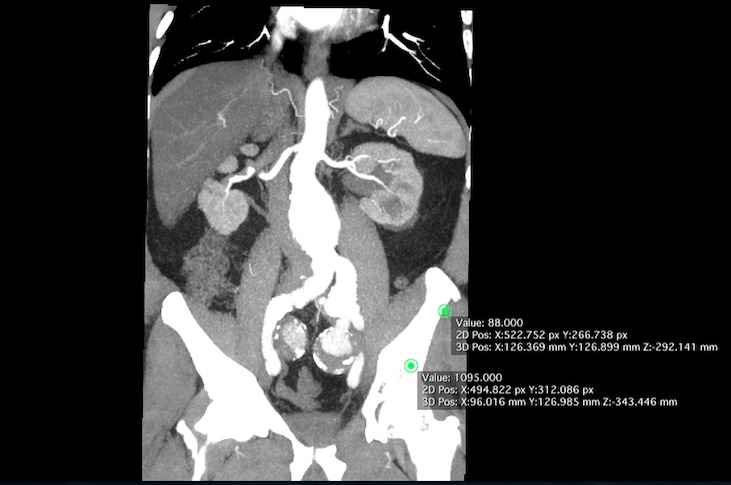
Angiotomografia pré-operatória em corte coronal.

Homem, 70 anos, hipertenso de longa data e assintomático. Foi diagnosticado com aneurisma de aorta abdominal por tomografia abdominal na pesquisa de neoplasia de próstata e então encaminhado ao serviço de referência. Realizou angiotomografia *multislice* que evidenciou aneurisma de aorta abdominal infrarrenal com maior diâmetro de 5,8 cm associado a aneurisma de artéria ilíaca comum esquerda e volumosos aneurismas de ambas artérias ilíacas internas, lado direito com diâmetro de 3,5 cm e lado esquerdo de 3,9 cm (Figuras 1 e 2).

**Figura 2:**
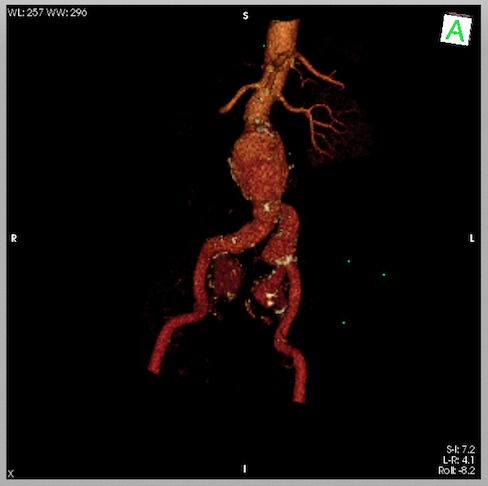
Angiotomografia pré-operatoria em reconstrução 3D.

Foi realizada intervenção eletiva após customização da endoprótese, com correção endovascular dos aneurismas com implante de endoprótese bifurcada Braile® (Braile Biomédica, São José do Rio Preto - Brasil) com módulo ramificado ilíaco esquerdo e embolização do aneurisma da artéria ilíaca interna direita com molas de liberação livre Braile® (Braile Biomédica, São José do Rio Preto - Brasil) e extensão do segmento ilíaco direito da endoprótese até a artéria ilíaca externa.

**Figura 3:**
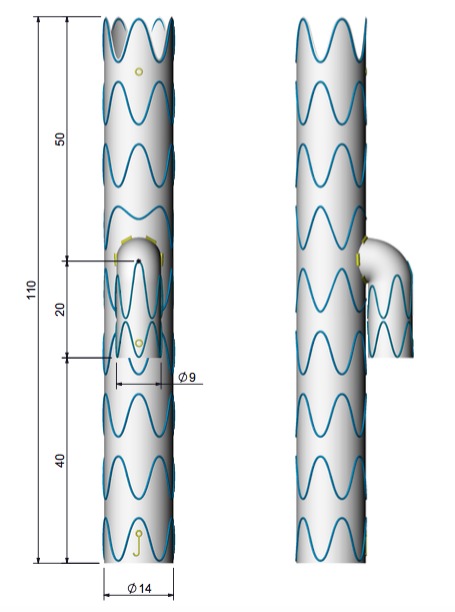
Projeto inicial de customização de endoprótese ramificada ilíaca.

A endoprótese ramificada foi customizada em dois modelos idênticos, sendo um para avaliação da liberação *ex vivo* pela equipe médica e com sistema de pré-cateterização do ramo ilíaco interno com cateter 4F e fio guia metálico 0,035” x 260 cm. Todo o processo de customização, incluindo testes, teve a duração de 20 dias. O projeto final somente foi liberado para fabricação após avaliação e consentimento da equipe médica (Figuras 3 e 4).

**Figura 4:**
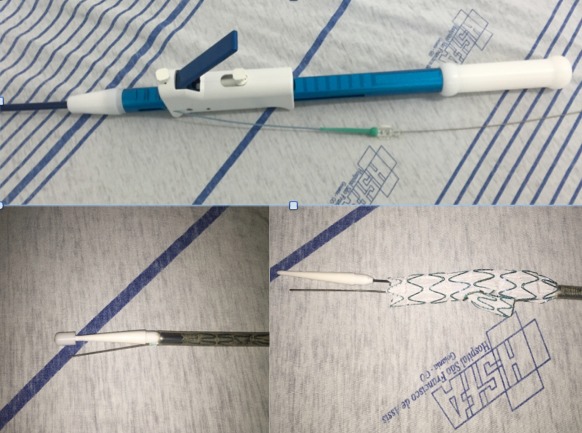
Apresentação em bancada da endoprótese com ramo ilíaco customizada e sistema de pré-cateterização para liberação em teste *ex vivo*.

O procedimento cirúrgico foi realizado em setor de hemodinâmica e com o paciente sob anestesia geral. Foram utilizados os acessos femorais e axilar esquerdo (todos por dissecção). Inicialmente foi realizada embolização do aneurisma da artéria ilíaca interna direita com molas de liberação livre medindo 32×15×15 mm, 32×10×10 mm e 32×8×8 mm seguida de implante de endoprótese com corpo principal de 26-14-170 mm e extensões ilíacas de 14x100 mm e 14×80 mm até a artéria ilíaca externa direita. A seguir, foi realizado cateterismo do ramo contralateral esquerdo, como habitual, implante de endoprótese ramificada ilíaca esquerda customizada de 14×110 mm e liberação parcial controlada até liberação do ramo ilíaco interno pré-cateterizado. Houve a passagem do fio guia pré-cateterizado até o arco aórtico e captura por laço pelo acesso axilar esquerdo (“técnica do varal”). Após, foi feita a introdução de bainha longa aramada 8F de 90 cm, pelo acesso axilar, com progressão até o ramo ilíaco interno da endoprótese e cateterismo do ramo glúteo esquerdo. Realizou-se então o implante de stent autoexpansível Fluency® (Bard Peripheral Vascular, Arizona/USA) de 9×100 mm, seguido de implante em seu interior de stent metálico E-luminexx® (Bard Peripheral Vascular, Arizona/USA) de 10×80 mm. Ao final, arteriografia de controle demonstrou exclusão completa dos aneurismas, manutenção do fluxo da artéria ilíaca interna esquerda e ausência de vazamentos. Foi utilizado total de 170 mL de contraste não iodado isosmolar.

Evolução pós-operatória ocorreu sem intercorrências em unidade de terapia intensiva e o paciente recebeu alta hospitalar no terceiro dia de internação sem complicações locais ou sistêmicas.

**Figura 5:**
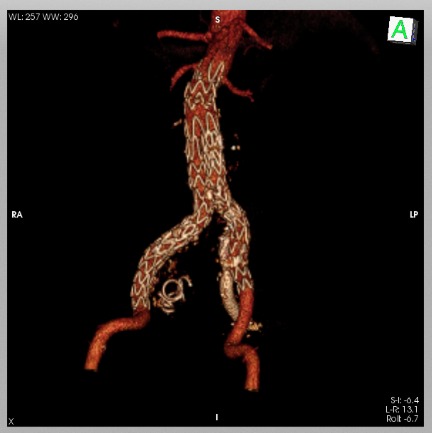
Angiotomografia de controle em reconstrução 3D.

Paciente encontra-se em acompanhamento ambulatorial com 7 meses de pós-operatório assintomático, sem complicações. Foram realizadas angiotomografias de controle com 1 mês, 3 meses e 6 meses, nas quais se observou manutenção da exclusão dos aneurismas, ausência de vazamentos e manutenção de fluxo no ramo ilíaco interno esquerdo (Figuras 5 e 6).

**Figura 6:**
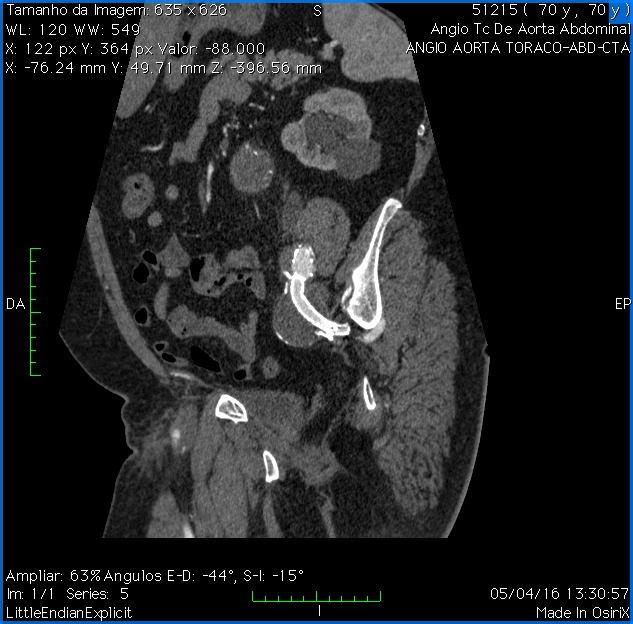
Angiotomografia de controle em conte sagital mostrando a exclusão do aneurisma da artéria ilíaca interna esquerda e manutenção da perviedade do ramo ilíaco.

## DISCUSSÃO

O tratamento dos aneurismas aortoilíacos vem evoluindo com o tempo^15^. A associação do aneurisma de aorta abdominal com o aneurisma das artérias ilíacas, principalmente das artérias ilíacas internas, torna o tratamento endovascular um desafio ainda maior. Inicialmente, técnicas de revascularização aberta eram realizadas na tentativa de evitar ou minimizar as complicações secundárias à isquemia pélvica; porém, havia um aumento significativo da morbidade. A embolização sequencial das artérias ilíacas internas é utilizada para ampliar a indicação do tratamento endovascular dos aneurismas aortoilíacos em anatomias desafiadoras, mas apresenta índices significativos de claudicação glútea e disfunção erétil^16^. Há uma tendência atual em preservar o fluxo pélvico no tratamento dos aneurismas aortoilíacos, mesmo quando há envolvimento das artérias ilíacas internas, e diretrizes específicas do manejo e tratamento da doença aneurismática aortoilíaca sugerem a preservação de pelo menos um ramo ilíaco interno^17^^,^ 18. Estudos vêm mostrando bons resultados em relação à manutenção do fluxo em ramo ilíaco, com sucesso técnico e baixa morbidade referente ao procedimento^10^^,^ 11 ^,^ 19 ^,^ 20.

A maioria das complicações importantes descritas após o implante desses dispositivos referem-se à oclusão aguda do ramo ilíaco. Apesar dessa incidência ser relativamente baixa, há relatos de complicações gravíssimas e fatais^7^, demostrando a necessidade de uma avaliação multidisciplinar, indicação restrita e utilização desses dispositivos por equipe médica treinada.

A evolução tecnológica dos dispositivos ramificados, bem como dos stents revestidos utilizados como ponte (*bridging stents*), associados a um planejamento pré-operatório criterioso, vem ajudando a atingir o objetivo de excluir os aneurismas aortoilíacos preservando a circulação pélvica e, dessa forma, minimizando os riscos de claudicação glútea, isquemia intestinal e impotência. Porém, o desafio terapêutico segue presente e uma porcentagem significativa desses pacientes ainda necessitará da oclusão das artérias ilíacas internas para tratamento definitivo dos aneurismas aortoilíacos.

## CONCLUSÃO

A preservação da circulação pélvica no tratamento endovascular do aneurisma aortoilíaco é objetivo de estudo em todo o mundo. A utilização de endopróteses com ramo ilíaco vem se mostrando importante opção terapêutica, e a indústria brasileira também busca produzir dispositivos IBD para auxiliar no tratamento dessa complexa patologia. O relato de caso apresenta bom resultado inicial com a primeira endoprótese brasileira com ramo ilíaco, porém estudos a longo prazo são necessários para conclusões em relação à utilização desses dispositivos.
