# Supplementary material for: Tratamento endovascular do aneurisma aortoilíaco: relato do primeiro caso utilizando endoprótese brasileira com ramo ilíaco
Source: J Vasc Bras. 2017 Jan-Mar;16(1):63–7. [Article in Portuguese] doi: 10.1590/1677-5449.011116 (PMC5829696; doi:10.1590/1677-5449.011116)
Supplement: Translation

# Translation: Endovascular treatment of aortoiliac aneurysm: first report of a case using a Brazilian device with an iliac branch

**Authors:** Fábio Augusto Cypreste Oliveira, Carlos Eduardo de Sousa Amorelli, Fábio Lemos Campedelli, Davi Heckmann, Juliana Caetano Barreto, Maria Cunha Ribeiro Amorelli, Ana Flávia Guerra Campedelli, Philippe Moreira da Silva

**Affiliations:** 1 Hospital São Francisco de Assis, Serviço de Angiologia, Cirurgia Vascular, Endovascular e Laserterapia – AngioGyn, Goiânia, GO, Brazil.; 2 CENTERVASC-Rio, Cirurgia Vascular, Rio de Janeiro, RJ, Brazil.; 3 Universidade Federal de Goiás – UFG, Hospital das Clínicas, Infectologia, Goiânia, GO, Brazil.; 4 Instituto Nacional de Câncer – INCA, Hematologia e Hemoterapia, Goiânia, GO, Brazil.; 5 Hospital Araújo Jorge, Radioterapia, Goiânia, GO, Brazil.

**Keywords:** abdominal aortic aneurysm, endovascular procedures, iliac artery

## Abstract

Aortoiliac aneurysms are particularly challenging to treat using a totally endovascular approach, because embolization of the internal iliac arteries can cause major complications. Several conventional and endovascular surgical techniques have been described that offer preservation of at least one internal iliac branch and options for completely endovascular treatment include branched stentgrafts and the parallel grafts technique. Here, the authors report the first case of endovascular treatment with preservation of an internal iliac branch using a Brazilian iliac branch device.

## INTRODUCTION

Endovascular repair of abdominal aorta aneurysms has been adopted worldwide and every year the number of cases treated this way increases.^1^ Endovascular treatment of infrarenal aneurysms has produced good results in terms of aneurysm exclusion and reduction of surgical morbidity and mortality.^2^ However, when an abdominal aortic aneurysm and an aneurysm of the iliac arteries are both present, the degree of treatment difficulty increases and it is a challenge to achieve the objective of excluding aneurysms while maintaining pelvic circulation because of the need to avoid bilateral embolization of the internal iliac artery and the complications secondary to this occlusion.^3^ According to the literature, cases that require unilateral or bilateral embolization of internal iliac arteries progress to gluteal claudication in 16-55% of cases, followed by impotence in 10-17% of cases.^4^^,^ 5 More severe complications such as medullary/mesenteric ischemia or gluteal necrosis are rare, but can occur in 1-3% of bilateral embolization cases.^6^^,^ 7 Because of this, several different revascularization techniques have been designed to attempt to preserve pelvic circulation by maintaining flow through at least one of the internal iliac arteries, including revascularization with bypass,^8^^,^ 9 placement of endoprostheses with iliac branches,^10^^,^ 11 and techniques using endoprostheses in parallel, such as the sandwich technique described by Lobato.^12^

Endoprostheses with iliac branches, known as iliac branch devices (IBD) were developed with the objective of enabling totally endovascular treatment of aortoiliac aneurysms, with exclusion of aneurysms and maintenance of anterograde flow into the internal iliac arteries. The initial results from these devices are favorable and promising.^11^^,^ 13 ^,^ 14

The objective of this article was to describe the first case of endovascular treatment of aortoiliac aneurysm using a Brazilian endoprosthesis with an iliac branch.

## CASE DESCRIPTION

The patient was an asymptomatic 70-year-old male with long-term hypertension. He had been diagnosed incidentally with abdominal aortic aneurysm by abdominal tomography conducted to investigate prostate cancer and referred to a vascular service. Multislice angiotomography showed an infrarenal abdominal aortic aneurysm with a largest diameter of 5.8 cm, an aneurysm of the left common iliac artery, and voluminous aneurysms of both internal iliac arteries, with diameters of 3.5 cm on the right and 3.9 cm on the left (Figures 1 and 2).

**Figure 1:** Coronal preoperative angiotomography.

**Figure 2:** Preoperative angiotomography in 3D reconstruction.

An elective intervention was performed after customization of an endoprosthesis. Endovascular repair of the aneurysms was achieved with placement of a Braile® bifurcated endoprosthesis (Braile Biomédica, São José do Rio Preto, Brazil) with a left iliac branch module, embolization of the aneurysm in the right internal iliac artery with Braile® free release coils (Braile Biomédica, São José do Rio Preto, Brazil), and extension of the right iliac segment of the endoprosthesis to the external iliac artery.

Two identical models of the customized branched endoprosthesis were constructed, one of which was used by the treating team to evaluate release ex vivo, using a system for pre-catheterization of the internal iliac with a 4Fr catheter and a 0.035”×260 cm metallic guide wire. The total duration of the entire customization process, including tests, was 20 days. The final design was only signed off for fabrication after it had been assessed and approved by the medical team (Figures 3 and 4).

**Figure 3:** Initial design of customized endoprosthesis with iliac branch.

**Figure 4:** Bench set up of customized endoprosthesis with iliac branch and pre-catheterization system for deployment in ex vivo test.

The surgical procedure was conducted in a hemodynamic suite with the patient under general anesthesia. Femoral and left axillary accesses were obtained (all by dissection). Initially, embolization of the right internal iliac artery aneurysm was achieved using free release coils measuring 32×15×15 mm, 32×10×10 mm, and 32×8×8 mm, followed by placement of an endoprosthesis with a 26-14-170 mm main body and iliac extensions of 14×100 mm and 14×80 mm, to the right external iliac artery. Next, catheterization of the left contralateral branch was conducted as normal and the 14×110 mm customized branched left iliac endoprosthesis was deployed with partial, controlled release until the pre-catheterized iliac internal branch was released. The pre-catheterized guide wire was advanced to the aortic arch and a noose used to perform capture via the left axillary access (“through-and-through technique”). Next, a 90 cm long, wired 8Fr sheath was introduced via the axillary access and advanced to the internal iliac branch of the endoprosthesis, followed by catheterization of the left gluteal branch. A 9×100 mm Fluency® self-expanding stent (Bard Peripheral Vascular, Arizona, USA) was deployed, followed by placement of a 10x80 mm E-luminexx® metallic stent (Bard Peripheral Vascular, Arizona, USA) inside it. At the end of these procedures, control arteriography showed complete exclusion of the aneurysms, preservation of flow into the left internal iliac artery, and absence of leaks. A total volume of 170 mL of non-iodinated iso-osmolar contrast was used.

Postoperative recovery in the intensive care unit was without intercurrent conditions and the patient was discharged from hospital on the third day after admission, with no local or systemic complications.

At the time of writing, the patient is asymptomatic, in outpatients follow-up, 7 months after the procedures and is free from complications. Control angiotomography was conducted at 1 month, 3 months, and 6 months, in all cases showing that aneurysms remained excluded, leaks were still absent, and flow through the left internal iliac branch was preserved (Figures 5 and 6).

**Figure 5:** Control angiotomography in 3D reconstruction.

**Figure 6:** Sagittal control angiotomography showing exclusion of left internal iliac artery aneurysm with preservation of iliac branch patency.

## DISCUSSION

Treatment of aortoiliac aneurysms has been evolving.^15^ The combination of abdominal aortic aneurysm with aneurysms of the iliac arteries increases the challenge of endovascular treatment, and even more so when it is the internal iliac arteries that are involved. Initially, open revascularization techniques were employed to try to avoid or minimize the complications secondary to pelvic ischemia; but there were significant increases in morbidity. Sequential embolization of the internal iliac arteries is employed to extend applications of endovascular treatment to aortoiliac aneurysms in challenging anatomies, but it is associated with significant rates of gluteal claudication and erectile dysfunction.^16^ The current tendency is to preserve pelvic flow when treating aortoiliac aneurysms, even when internal iliac arteries are involved, and specific directives for management and treatment of aneurysmal aortoiliac disease suggest that at least one internal iliac branch should be preserved.^17^^,^ 18 Studies have been published showing good results in terms of preservation of flow to an iliac branch and reporting technical success and low morbidity with the procedure.^10^^,^ 11 ^,^ 19 ^,^ 20

The majority of significant complications described after implantation of these devices are related to acute occlusion of the iliac branch. While incidence is relatively low, there are reports of extremely serious and even fatal complications,^7^ which underscores the need for multidisciplinary assessment, strict adherence to indications, and deployment of these devices by a trained medical team.

Technological developments in branched devices and bridging stents, combined with careful preoperative planning, are helping us to achieve the objective of excluding aortoiliac aneurysms while preserving pelvic circulation, thereby minimizing the risks of gluteal claudication, intestinal ischemia, and impotence. However, the therapeutic challenge still remains and in a significant percentage of these patients it will still be necessary to accept occlusion of internal iliac arteries to achieve definitive treatment of aortoiliac aneurysms.

## CONCLUSIONS

Preservation of pelvic circulation while achieving endovascular treatment of aortoiliac aneurysms is an objective of studies all over the world. Use of endoprostheses with an iliac branch is proving to be an important treatment option and the Brazilian medical industry is also working to produce IBDs to enable treatment of this complex pathology. The case described in this report shows a good initial result after use of the first Brazilian endoprosthesis with an iliac branch, but long-term studies will be needed to arrive at firm conclusions with relation to use of these devices.
